# Development and Validation of an Innovative Analytical Approach for the Quantitation of Tris(Hydroxymethyl)Aminomethane (TRIS) in Pharmaceutical Formulations by Liquid Chromatography Tandem Mass Spectrometry

**DOI:** 10.3390/molecules28010073

**Published:** 2022-12-22

**Authors:** Moran Madmon, Tamar Shamai Yamin, Shani Pitel, Chen Belay, Yaniv Segula, Einat Toister, Ariel Hindi, Lilach Cherry, Yakir Ophir, Ran Zichel, Avishai Mimran, Eran Diamant, Avi Weissberg

**Affiliations:** 1 Department of Analytical Chemistry, Israel Institute for Biological Research (IIBR), P.O. Box 19, Ness Ziona 7410001, Israel; 2 Department of Biotechnology, Israel Institute for Biological Research (IIBR), P.O. Box 19, Ness Ziona 7410001, Israel

**Keywords:** TRIS, tromethamine, pharmaceuticals, COVID-19 vaccine, derivatization, isobutyl chloroformate, quantitation, LC-MS, validation, GMP

## Abstract

A novel COVID-19 vaccine (BriLife^®^) has been developed by the Israel Institute for Biological Research (IIBR) to prevent the spread of the SARS-CoV-2 virus throughout the population in Israel. One of the components in the vaccine formulation is tris(hydroxymethyl)aminomethane (tromethamine, TRIS), a buffering agent. TRIS is a commonly used excipient in various approved parenteral medicinal products, including the mRNA COVID-19 vaccines produced by Pfizer/BioNtech and Moderna. TRIS is a hydrophilic basic compound that does not contain any chromophores/fluorophores and hence cannot be retained and detected by reverse-phase liquid chromatography (RPLC)-ultraviolet (UV)/fluorescence methods. Among the few extant methods for TRIS determination, all exhibit a lack of selectivity and/or sensitivity and require laborious sample treatment. In this study, LC–mass spectrometry (MS) with its inherent selectivity and sensitivity in the multiple reaction monitoring (MRM) mode was utilized, for the first time, as an alternative method for TRIS quantitation. Extensive validation of the developed method demonstrated suitable specificity, linearity, precision, accuracy and robustness over the investigated concentration range (1.2–4.8 mg/mL). Specifically, the R^2^ of the standard curve was >0.999, the recovery was >92%, and the coefficient of variance (%CV) was <12% and <6% for repeatability and intermediate precision, respectively. Moreover, the method was validated in accordance with strict Good Manufacturing Practice (GMP) guidelines. The developed method provides valuable tools that pharmaceutical companies can use for TRIS quantitation in vaccines and other pharmaceutical products.

## 1. Introduction

The COVID-19 pandemic caused by severe acute respiratory syndrome coronavirus 2 (SARS-CoV-2) has presented a major worldwide challenge. Recombinant vesicular stomatitis virus (rVSV) expressing the SARS-CoV-2 spike protein instead of the glycoprotein (G) of VSV [1] is the active pharmaceutical ingredient of a novel vaccine (BriLife^®^) that has recently been developed by the Israel Institute for Biological Research (IIBR) [2,3]. Phase II clinical trial findings are currently being analyzed. The final formulation of the drug product contains 20 mM (2.4 mg/mL) tromethamine (TRIS) to stabilize the drug substance, i.e., the rVSV-SARS-CoV-2-S viral vector. TRIS is a biologically inert polar amino alcohol with low toxicity that is extensively used in biochemistry and molecular biology as a component of buffer solutions [4]. TRIS has a pKa of 7.8 at 37 °C and is thus an effective buffer in the physiological range that can stabilize and prevent pH fluctuations in solution [4]. TRIS is a solid readily soluble in water [5] and its physicochemical properties have been broadly reviewed [6]. TRIS plays a very important role in vaccine formulations (i.e., the Moderna vaccine [7,8,9] and the updated Pfizer/BioNtech COVID-19 vaccine) [10,11]. The use of TRIS as a buffer enables the stabilization of a product and extends the product shelf life by maintaining the pH. TRIS acts as an osmotic diuretic, increasing urine flow, urinary pH, and excretion of fixed acids, carbon dioxide and electrolytes [11]. Furthermore, TRIS is commonly used as an emulsifying agent in pharmaceutical and cosmetic products or as a counterion for acidic pharmaceutical compounds to generate desired salt forms [12,13,14,15,16]. Suitable quality control analytical methods are required to quantitate TRIS for regulatory purposes. The European [17] and US [18] pharmacopeia describe a simple titration against HCl for assay purposes. However, this method is not sufficiently sensitive, requires a high sample volume and concentration, and suffers from hindrance effects in complex buffered formulations; therefore, a separative method may be a more effective alternative. To date, only a few chromatography-based methods have been developed for TRIS determination in pharmaceutical products and biological fluids. Two reverse-phase (RP) HPLC methods involving chemical derivatization have been developed that provide sufficient retention, as well as chromophores or fluorophores for UV/fluorescence detection [13,19]. Naidong and Ghodbane determined TRIS as an excipient in Iopamidol injection (a formulated pharmaceutical product) by derivatization and LC-UV analysis at a concentration of 1.0 mg/mL [20]. Other methods include ion chromatography (IC) followed by conductivity detection [12] and a capillary electrophoresis (CE)-based method [14]. An alternative approach to realize sufficient retention of the polar TRIS compound is to use hydrophilic interaction chromatography (HILIC) as a normal phase prior to analysis with a refractive index (RI) detector [15]. More recently, HILIC has been coupled with a charged aerosol detector [21]. Our initial attempts to develop an assay based on HPLC coupled with a UV detector with and without derivatization required complex sample treatment (two-step derivatization, extractions and evaporation to dryness) and resulted in low performance.

The current study is the first development of a simple, rapid, sensitive, specific, and reproducible RP-LC– electrospray ionization (ESI) tandem mass spectrometry (MS/MS)-based method to determine TRIS concentrations after derivatization in a vaccine formulation. The method was further validated for the Israeli BriLife^®^ vaccine according to the requirements of the International Council for Harmonisation (ICH) under Good Manufacturing Practice (GMP) guidelines. This novel GMP-compliant RP-LC–ESI-MS/MS method is thus a vital tool for pharmaceutical companies to determine TRIS concentrations in other vaccines and pharmaceutical formulations.

## 2. Results and Discussion

### 2.1. RP-LC–MS/MS Strategy

TRIS is a small organic compound that contains three aliphatic hydroxyl groups and a primary amine. An initial study on TRIS derivatization with benzoyl chloride (a chromophore-containing molecule) followed by UV detection revealed that the matrix components (e.g., NaCl, trehalose, rHSA) caused peak broadening that masked the TRIS derivative, creating difficulties with the quantitation assay (data not shown). In contrast to UV/fluorescence detection, MS detection does not require the presence of a chromophore. However, chromatography of TRIS in the C18 RP columns is challenging due to interactions between the amine group and the free silanol groups attached to the stationary phase. Initial experiments revealed that a nonretainable, broad peak of intact TRIS was observed under RPLC–MS conditions. To overcome this difficulty and improve the chromatographic retention and peak shape in RP-LC–ESI-MS/MS, a novel method based on chemical derivatization was developed. RP-LC–ESI-MS/MS detection was far more sensitive than UV/fluorescence detection; in addition, the relatively high TRIS concentration in the vaccine formulation (2.4 mg/mL) enabled the replacement of a required clean-up step prior to UV/fluorescence analysis by a simple high-dilution step of the sample with an organic solvent (acetone). Dilution with acetone rather than with an aqueous medium was also more suitable for the derivatization reaction (carbamylation). After the derivatization step and prior to analysis, a second high-dilution of the sample with water was required for the sample concentration to fall into the linear range of the MS detector and to improve the RP-LC–ESI-MS/MS analysis results. This second dilution step has the advantages of eliminating ion suppression or enhancement caused by either the matrix or the derivatizing agent that may affect the RP-LC–ESI-MS/MS quantitation.

**Figure 1:**
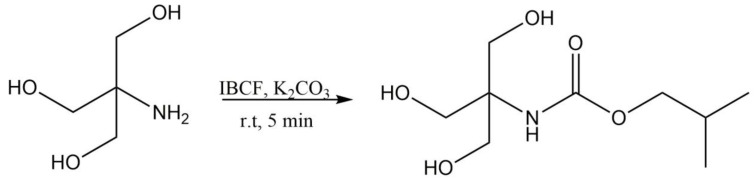
Scheme of the reaction of Tris(hydroxymethyl)aminomethane (TRIS) with IBCF.

A commonly used chemical reaction for reducing the reactivity and polarity of an amine nitrogen is the formation of a carbamate protecting group. Therefore, rapid and selective derivatization was conducted with IBCF to form a stable carbamate derivative of TRIS. The primary amine reacts readily with IBCF, while the hydroxyl groups remain intact. The reaction scheme of TRIS with IBCF is shown in Figure 1.

### 2.2. Assay Development

#### 2.2.1. Chromatographic Parameters

**Figure 2:**
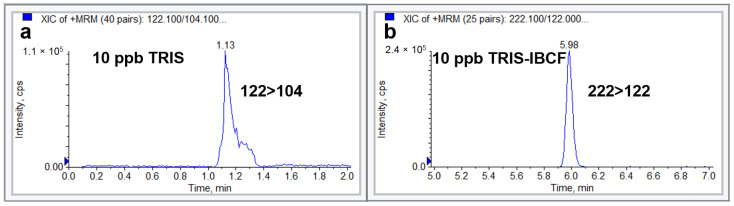
The chromatographic peak shape of TRIS before (**a**) and after derivatization (**b**) with IBCF at 10 ng/mL.

A 1 µg/mL TRIS solution in acetone was derivatized with IBCF at 50 °C for 30 min, diluted 1:10 with water and analyzed by RP-LC–ESI-MS/MS. We utilized a chromatographic method that was recently developed for carbamate derivatives of phenidate analogues [22]. The chromatographic conditions were as follows: a RP C18 Gemini Phenomenex column and mobile phase compositions of water (pH = 6.4) and methanol containing 1 mM ammonium formate. Three parameters were initially analyzed using the chromatographic data: the retention time (Rt), peak height (H), and peak width at half height. A proper peak shape (4-sec width at half height) with a relatively high intensity signal was achieved for the TRIS derivative (TRIS-IBCF) using neutral elution solvents, in contrast to a nonretainable and broad peak for underivatized TRIS. Figure 2 shows the chromatographic peak shape of TRIS prior to and following derivatization. Derivatization reduced the polarity, resulting in improvement of the carbamate peak shape and extension of the carbamate retention time in RP-LC. As a suitable retention time and symmetrical peak shape were obtained under these conditions, no other chromatographic conditions were investigated.

#### 2.2.2. Structurally Informative RP-LC-ESI-MS/MS

EPI (MS/MS) experiments were carried out on the derivatization product (TRIS-IBCF) at various collision energies (10–50 eV). The ESI-MS/MS spectra of TRIS-IBCF were interpreted and characterized, and the MRM transitions were identified.

**Figure 3:**
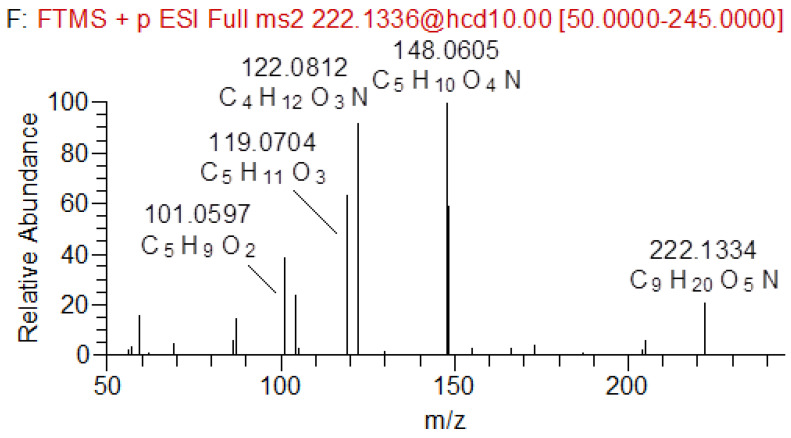
Orbitrap-ESI-MS^2^ spectra of TRIS-IBCF.

The positive ESI-MS/MS spectrum of TRIS derivatized with IBCF contained several product ions at *m/z* 204, 148, 122, 119, 104 and 101. Fragmentation pattern of protonated TRIS-IBCF and plausible ion structures are depicted in Figure S1. One of the two most intense product ions in the ESI-MS/MS spectrum was observed at *m/z* 148 and corresponded to the loss of isobutanol from the [M + H]^+^ 222 precursor ion. The second of the most intense product ions was at *m/z* 122 (intact TRIS), generated by a loss of the isobutyl formate moiety. These two specific MRM transitions (*m/z* 222→122 and *m/z* 222→148) had a signal intensity ratio of ~1:3.5 and a defined retention time (RT = 5.98 min) and were selected for detection and verification. All the observed QTRAP-MS/MS (MS^2^) product ions (Figure S1) were supported by Orbitrap-ESI-MS^2^ exact mass measurements (<3 ppm error), enabling detailed structural elucidation of the TRIS-IBCF derivative. The Orbitrap-ESI-MS^2^ spectrum of the TRIS-IBCF derivative is shown in Figure 3.

#### 2.2.3. Optimization of the Derivatization Reaction

Several derivatization reaction parameters were investigated and optimized: the reaction temperature (25 and 50 °C), reaction time (5 and 30 min), solvent (acetone and water) and IBCF derivatizing agent concentration (1–4 mg/mL). A constant K_2_CO_3_ concentration (1 mg/mL) was utilized in all experiments. The influence of all the parameters on the conversion of TRIS to the corresponding carbamate counterpart was studied. Preliminary results demonstrated higher conversion yields (>98% in comparison to ~10% in water) and reaction rates using an organic solvent (acetone); therefore, acetone was selected as the solvent of choice. Initial experiments revealed no effect of heating on the derivatization yield of TRIS. TRIS was converted to its carbamate derivatives within 5 min at ambient temperature. N-isobutyl carbonylation times of TRIS longer than 5 min resulted in a constant signal intensity. Increasing the reaction temperature to 50 °C for 30 min did not change the signal intensity of the carbamate counterpart. Despite the rather high base concentration used (1 mg/mL), no ion suppression was observed because the sample was highly diluted prior to RP-LC–ESI–MS/MS analysis. To determine the minimum concentration of the derivatizing agent necessary for quantitative conversion to yield a high-intensity signal, the reaction was studied under a reduced IBCF concentration. Reducing the IBCF concentration from 4 mg/mL to 1 mg/mL resulted in a decreased conversion yield after a 5 min reaction. Based on these optimization results, the following derivatization protocol was used: a 4 mg/mL derivatizing agent (IBCF), 1 mg/mL K_2_CO_3_, a reaction time of 5 min at room temperature, and acetone as a solvent. Notably, TRIS derivatized in standard solutions, matrix-like solutions (MTRX) and the vaccine formulations after 1:500 dilution with acetone resulted in the same peak area as TRIS derivatized in pure acetone. Therefore, all blanks, standards and vaccine formulations were diluted by 1:500 with acetone prior to carrying out the derivatization reaction.

#### 2.2.4. Dilution of the Derivatization Reaction Mixture Prior to LC–ESI–MS/MS Analysis

Preliminary results demonstrated that dilution of the reaction mixture by 1:500 with water prior to RP-LC–ESI–MS/MS analysis resulted in good chromatographic results and a signal in the linear range of the detector (0.1- 30 ng/mL). However, slight degradation of the derivatization product was observed over time. We speculated that an excess of derivatizing agent may have also reacted with the hydroxyl groups of the derivatization product. IBCF is hydrolyzed in water to generate alcohol, carbon dioxide, and hydrogen chloride. However, hydrolysis is more rapid in water containing acid. Therefore, the dilution of the derivatization reaction mixture prior to analysis was performed in an aqueous solution containing 0.1% formic acid to decompose the excess IBCF derivatizing agent. Indeed, dilution with aqueous solution containing 0.1% formic acid led to a very stable derivatization product. Stability data of the derivatized product are provided in the section on robustness (2.3.6).

### 2.3. Assay Validation

After the test procedure was established, a validation protocol was designed to provide sufficient evidence that the analytical procedure met the objectives and was suitable for TRIS quantitation. Validation was performed according to the requirements of the International Council for Harmonisation (ICH) Q2 (R1) concerning validation of analytical procedures [23] under Good Manufacturing Practice (GMP) conditions. These requirements include specificity, linearity, range, accuracy, precision, robustness, and system suitability. Although LC–MS/MS-based methods enable TRIS determination at concentrations considerably below mg/mL levels, such low concentrations are not within the TRIS concentration range in the vaccine formulation and therefore were not investigated. The quantification limit was practically set as the lowest TRIS concentration (1.2 mg/mL) determined from the calibration curve, as validated by goodness-of-fit (R^2^) and back-calculation data during the linearity and range analysis.

#### 2.3.1. Specificity

**Figure 4:**
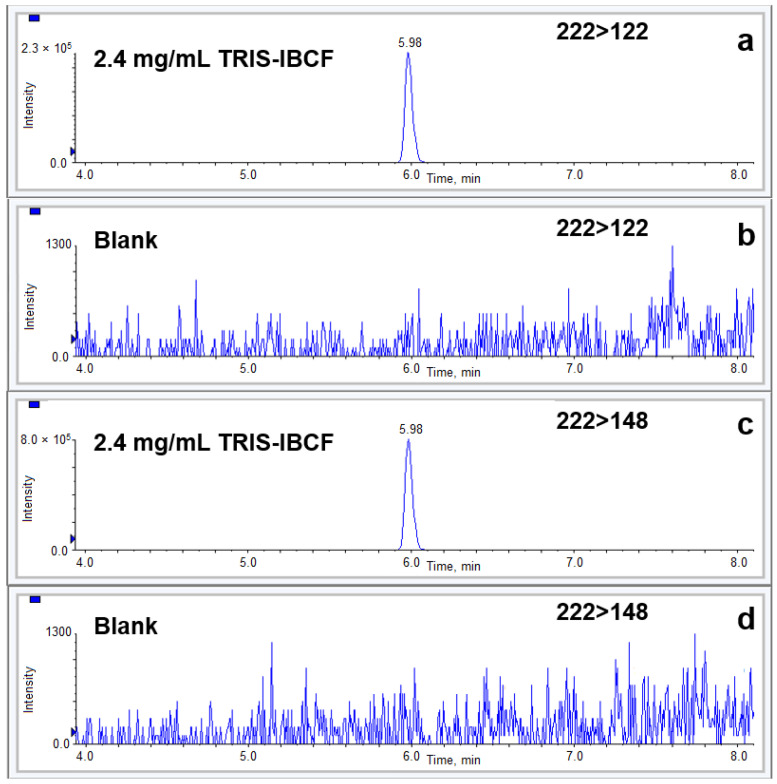
Representative MRM chromatograms of 2.4 mg/mL TRIS-IBCF in the vaccine formulation: a quantifier MRM chromatogram 222→122 (**a**) and a qualifier MRM chromatogram 222→148 (**c**) along with a blank MTRX (vaccine formulation without Tris ((**b**,**d**), corresponding to (**a**,**c**)).

The specificity of the analytical method was evaluated based on the results of an identification test, determination of impurities and the assay. The identification test results showed that TRIS-IBCF could be discriminated from other compounds in the matrix. That is, positive results using the MTRX spiked with TRIS were obtained in parallel with negative results using the MTRX alone. The two most dominant MRM transitions were selected for quantitative and confirmative analyses. The MRM transition MH^+^→122 (quantifier transition) was used for quantitative analysis. The MRM transition MH^+^→148 (qualifier transition) was utilized to confirm the identification result. Considering the performance criteria of the EU Commission [24], the MRM ratio between the abundances of the two selected precursor and product-ion transitions (the quantifier transition to the qualifier transition) was used to confirm the analyte identification. The commonly used European criteria for identification substantially minimize the potential for false-positives due to similar reaction chemistry. Additionally, assay and impurity tests were conducted to rule out bias of the results by determining the TRIS concentration in vaccine batches containing other excipients, including the virus. Three different vaccine preparations were assayed, and the TRIS concentrations were found to be 2.50, 2.42, and 2.39 mg/mL. These results were very close to the expected 2.4 mg/mL weighed in all vaccine preparations, and the deviations, expressed as relative errors (%RE), were only 4.3%, 0.6%, and −0.3% for the aforementioned concentrations. The preparations contained no interferences from other components in the analyte MRM window. Figure 4 shows a typical quantifier MRM chromatogram (222→122) of a sample spiked at a concentration of 2.4 mg/mL along with blank MTRX (containing all the added excipients except TRIS) after applying the derivatization procedure. An excellent peak shape (a half-height peak width of 4.4 s) for TRIS-IBCF was observed with a suitable retention time (5.98 min), which was sufficiently long to prevent matrix effects but not too long to produce a reasonable cycle time of 12 min. A typical qualifier MRM chromatogram (222→148) of a sample along with a blank was also demonstrated. The matrix effect was eliminated by two high dilutions of 1:500 with acetone prior to derivatization and 1:500 with aqueous 0.1% formic acid.

#### 2.3.2. Linearity

**Figure 5:**
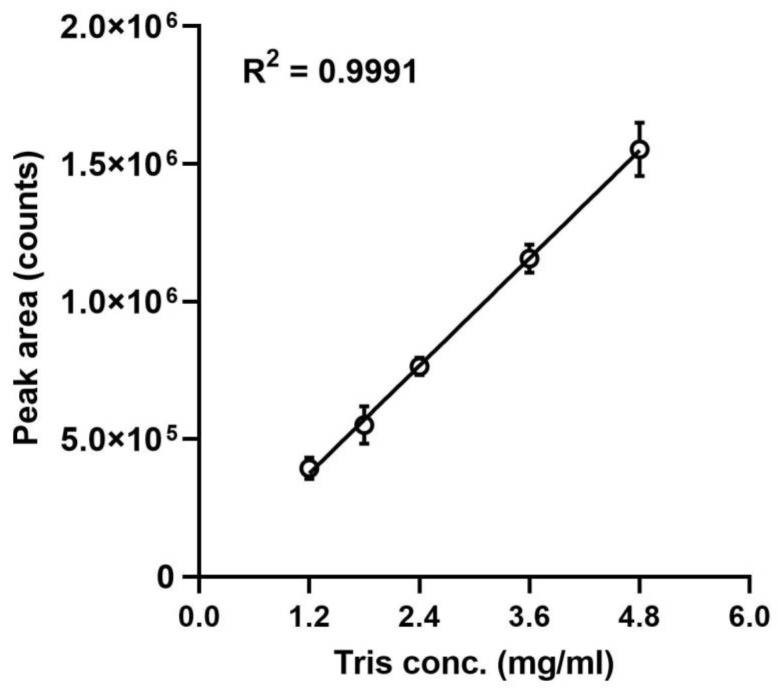
A five-point calibration curve. The peak areas were plotted against the TRIS-IBCF concentrations. The regression line was calculated for concentrations of triplicates of independent derivatizations of a standard TRIS solution. Error bars are ± 1 standard deviation.

The linearity was determined by calculating the regression line of a five-point calibration curve using the method of least squares. The goodness-of-fit (R^2^), % y-intercept, and slope of the regression line were assessed for 5 concentrations of standard TRIS solutions ranging from 1.2 to 4.8 mg/mL, thereby covering the expected target concentration in the vaccine preparation (2.4 mg/mL). Each of the test concentrations was determined by three independent derivatizations. Curves were constructed by plotting the peak area against the TRIS-IBCF concentration (Figure 5). The goodness-of-fit R^2^ was 0.9991, and the slope was 325953. In addition, the % y-intercept was calculated by dividing the value at which the regression line intersected the y-coordinate by the peak area obtained for a 2.4 mg/mL concentration and found to be −2.1%. Back-calculating the concentrations from the regression line resulted in deviations of 4.7%, −3.4%, −0.2%, −0.1%, and 0.3% from the known concentrations of the calibration curve of 1.2 mg/mL, 1.8 mg/mL, 2.4 mg/mL, 3.6 mg/mL, and 4.8 mg/mL, respectively.

#### 2.3.3. Accuracy

A four-level spiking approach was used to determine accuracy, i.e., the closeness of agreement between the expected and measured TRIS concentrations. Twelve determinations over four concentration levels were conducted (three replicates for each of the four concentrations, in which each replicate was determined based on three independent derivatizations), covering 50–125% of the expected 2.4 mg/mL TRIS concentration in the vaccine preparation. To assess the accuracy, one volume of a standard solution containing 0% (MTRX only), 50% (1.2 mg/mL TRIS), 100% (2.4 mg/mL TRIS), and 150% (3.6 mg/mL TRIS) of TRIS was added to one volume of a vaccine preparation, and the recovery of the quantity of TRIS was calculated. The recovery of TRIS in the standard/vaccine mixtures ranged between 92% and 105% (Table 1), indicating that quantification of TRIS by the derivatization-RP-LC–ESI-MS/MS method was accurate.

#### 2.3.4. Precision

The precision of the method was analyzed to determine the degree of scatter between a series of measurements obtained from multiple samplings of the same homogeneous QC samples and vaccine preparations. Precision, resolved as repeatability and intermediate precision, was expressed as the coefficient of variation in percentages (%CV) of a series of measurements (TRIS concentrations and retention times).

##### Repeatability

Repeatability, also termed intra-assay precision, expresses the precision under the same operating conditions over a short interval of time [23]. The repeatability of the analytical method was determined by conducting six independent derivatizations for vaccine preparation and measuring the resulting %CV of both the six TRIS-IBCF concentrations and retention times. In all three experiments used to test the method repeatability, the calculated %CV values for the concentrations and retention times were 5.2–11.9% and 0.07–0.09%, respectively (Table 2 and Table S1). In addition to the intra-assay precision of the derivatizations, repeatability of the RP-LC–ESI-MS/MS performance was also tested. That is, the same TRIS-IBCF derivate was run six times, and the %CV of the resulting concentrations and retention times was calculated. In all the experiments, the %CV of the multiple runs of a single TRIS-IBCF derivate ranged between 1.2% and 1.7%, and the %CV of Rt was 0.00–0.09% (Table 2 and Table S2).

##### Intermediate Precision

Intermediate precision is a measure of interassay variations. Factors to be considered are potential sources of variability, for example, experimental days, environmental conditions and analysts [23]. To assess the intermediate precision, measurements of the same vaccine preparation and the same QC sample made on three different days by two different analysts were compared. Table 3 shows that the %CV of the TRIS concentrations was only 5.6% for the vaccine preparation, and 3.8% among different days for the QC sample.

#### 2.3.5. Range

The range was established by confirming that the analytical procedure provided an acceptable degree of linearity, accuracy and precision when applied to standard solutions containing TRIS concentrations within 75–125% of the expected concentration in a vaccine preparation. In addition to the data on linearity, accuracy, and precision in the specified range that had already been obtained, more data were obtained by analyzing the repeatability of TRIS measurements at the target concentration and the extremes of the range from both linearity and accuracy studies. As all measurements were conducted in triplicate, the %CV of TRIS concentrations at 1.8 mg/mL (75% of the target), 2.4 mg/mL (100% of the target), and 3.0 mg/mL (125% of the target) could be calculated (Table 4).

Repeatability data from linearity and accuracy determination showed that the %CV values of the TRIS measurements did not exceed 10.1% within the 75–125% of the specified range. This analysis supported the results of the linearity, accuracy, and precision studies detailed in Section 2.3.2, Section 2.3.3 and Section 2.3.4. Altogether, our data demonstrated that the novel RP-LC-ESI–MS/MS method has a suitable level of linearity, accuracy, and precision within the required concentration range of 75–125% of the target TRIS concentration in the BriLife^®^ vaccine.

#### 2.3.6. Robustness

The robustness of the novel RP-LC-ESI-MS/MS-based TRIS quantitation method to small variations in several method parameters was tested to obtain an indication of the method reliability during normal usage. Robustness was evaluated by slightly modifying the column temperature and flow rate that may affect selectivity, resolution, and sensitivity and by using a column from a different batch. A routine system suitability check was performed, and TRIS measurements (peak areas) in a QC sample were made under normal conditions. Thereafter, each parameter was changed deliberately, one at a time, while keeping other variables constant, and the repeatability and accuracy of TRIS measurements on the same QC sample were determined under the modified conditions. Table 5 presents the results for the QC sample when the column temperature was modified by 2 °C (5.0%, from 40 °C to 38 °C and to 42 °C), the flow rate was changed by 0.003 mL/minutes (1.0%, from 0.300 to 0.297 and 0.303 mL/minutes), and the column was replaced by another column.

**Figure 6:**
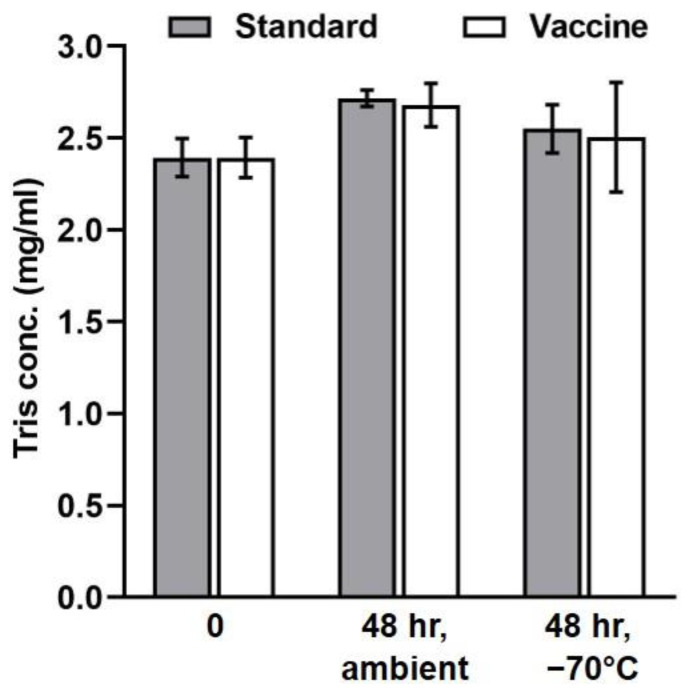
Stability of TRIS-IBCF at ambient temperature for 48 hr. A standard solution and a vaccine preparation were derivatized in triplicate. The derivates were split into two equal samples stored at −70 °C and ambient temperature. The derivates were then analyzed by RP-LC-ESI-MS/MS, and the TRIS concentration in these samples was determined both at zero time and after 48 h. Error bars are ± 1 standard deviation.

In addition to making deliberate small variations in the method parameters, the TRIS stability in the standard solution and a vaccine preparation was also investigated during the robustness studies. To evaluate the chemical stability of the TRIS derivative, the standard solution (2.4 mg/mL TRIS) and vaccine preparation (the drug formulation containing 2.4 mg/mL TRIS) were prepared (derivatized) in triplicate and analyzed by RP-LC–ESI–MS/MS. Then, each derivate was split into two equal samples stored at −70 °C and ambient temperature. The TRIS concentration of these samples was determined both at zero time and after 48 hr (Figure 6). The TRIS-IBCF was highly stable in both the standard solution and vaccine preparation at ambient temperature for 48 hr, as evidenced by relative errors between the TRIS-IBCF concentrations at ambient temperature and −70 °C of only 6.6% and 4.9%, respectively. 

#### 2.3.7. System Suitability

The reproducibility of the LC-auto sampler injector and MS detector (LC–MS system) was determined by performing six successive injections of a 2.4 mg/mL standard solution of TRIS, followed by a single injection of the same standard solution at the end of the experimental day. Analysis of the data from all the experiments demonstrated a relative standard deviation (RSD) of <5% among the daily series of six successive injections (Table 6 and Table S4). Additionally, the deviation between the average result of the six injections and the last single injection was less than 10% (Table 6 and Table S4).

## 3. Materials and Methods

### 3.1. Chemicals and Reagents

Acetone, ammonium formate, potassium carbonate, IBCF, TRISMA base (TRIS), rHSA (recombinant human serum albumin), sodium chloride, and trehalose were purchased from Merck. Water (MS grade), methanol (MS grade) and formic acid (MS grade) were purchased from Biolab (Jerusalem, Israel)

### 3.2. Stock Solution, Standard Solutions, Quality Control Samples, and Vaccine Formulation

A matrix-like solution (MTRX), resembling the BriLife^®^ vaccine preparation excluding TRIS, was prepared by dissolving 250 mg of recombinant human serum albumin (rHSA), 900 mg of NaCl, and 4 g of trehalose in 100 mL of purified water. The solution was vortexed for a sufficient period of time to achieve complete homogenization.

A 12 mg/mL TRIS stock solution was prepared in a volumetric flask by dissolving 240 mg of TRIS in 20 mL of MTRX.

Standards were prepared by diluting the TRIS stock solution in MTRX to concentrations of 1.2, 1.8, 2.4, 3.6, and 4.8 mg/mL. Different TRIS concentrations were prepared to generate a standard curve as follows: 1.0, 1.5, 2.0, 3.0, and 4.0 mL of 12 mg/mL TRIS were transferred to 10-mL volumetric flasks, and a MTRX solution was added to produce final volumes of 10 mL, resulting in TRIS concentrations of 1.2, 1.8, 2.4, 3.6, and 4.8 mg/mL, respectively.

TRIS quality control (QC) samples, at a concentration of 2.4 mg/mL, were prepared by dissolving 250 mg of rHSA, 900 mg of NaCl, 4 g of trehalose, and 240 mg of TRIS in 100 mL of purified water. The solution was vortexed for a sufficient period of time to achieve complete homogenization.

A vaccine formulation was prepared according to Makovitzki et al. [2] and Lerer et al. [3]. Briefly, the vaccine is based on an rVSV-SARS-CoV-2-S replicating virus. The virus is produced in Vero cells grown on Fibra-Cel bioreactors [25] containing serum-free medium. At the end of the upstream process, the culture medium with the virus is collected. A multistep downstream process comprising sequential endonuclease digestion, depth and membrane filter clarification, and chromatographic purification and ultrafiltration is used to rescue the virus from the medium and eliminate process- and product-related impurities (e.g., endonuclease, host-cell proteins, metabolites, and host-cell DNA). The final purified vaccine preparation is formulated with 2.5 mg/mL rHSA, 9.0 mg/mL NaCl, 40 mg/mL trehalose, and 2.4 mg/mL TRIS.

### 3.3. Liquid Chromatography–Mass Spectrometry (LC–MS)

#### 3.3.1. Liquid Chromatography

TRIS and TRIS-IBCF were separated using an Agilent 1290 HPLC system (Palo Alto, CA, USA), which consisted of a 1290 Infinity Binary Pump containing a Jet Weaver V35 Mixer, 1290 Infinity Autosampler and a 1290 Infinity Thermostatted Column Compartment.

HPLC conditions: Gradient elution was performed on a reverse-phase separation column (Geminutesi C18, 3.0 µM, 150 mm, 2.1 mm ID by Phenomenex, Switzerland) at a flow rate of 0.3 mL/minute. The column was maintained at 400 °C in all experiments. The mobile phase consisted of water (Solvent A) containing 1 mM ammonium formate and 5% MeOH and MeOH containing 1 mM ammonium formate (Solvent B). The following elution gradient was used: 0% B/100% A was converted to 95% B over 8 min, and the initial conditions were returned to over 4 min (0% B/100% A).

#### 3.3.2. Quadrupole Ion Trap (MS/MS and MRM Experiments)

Mass spectra of the analytes were acquired using an Applied Biosystems 5500 QTRAP linear ion-trap quadrupole mass spectrometer (AB Sciex, Foster City, CA, USA) with Analyst software (version 1.6.2, Woodlands, Singapore) equipped with a Turbo V ion source operated in the positive electrospray ionization (ESI) mode.

The ESI inlet conditions were as follows: Gas 1, nitrogen (40 psi); Gas 2, nitrogen (70 psi); ion spray voltage, 4500 V; ion source temperature, 60 °C; and curtain gas, nitrogen (35 psi).

Enhanced product ion (EPI) MS^2^ experiments-EPI scans were obtained using the following settings: the collision gas was set at “high”, and the collision energy was set between 10 and 50 eV. The fixed LIT fill time was set to 20 ms.

MRM experiments. The detection and identification of TRIS after derivatization with IBCF was performed in positive ion MRM mode using the two most abundant product ions: 222→148; CE 20 eV and 222→122; CE 25 eV. The quantifier transition of TRIS-IBCF was 222→122, and the 222–148 MRM transition was utilized as a qualifier transition to confirm the identification of TRIS-IBCF.

#### 3.3.3. Orbitrap-MS

A Thermo Scientific Orbitrap MS (Q-Exactive Plus; Thermo Fisher Scientific, Bremen, Germany) was operated with a heated ESI (HESI) source. The MS and MS/MS experiments were performed in positive ion mode.

The major Orbitrap HESI operating parameters were as follows: electrospray voltage, 1.25 kV; sheath gas flow rate, 45 (arbitrary units); auxiliary gas flow rate, 10 (arbitrary units); sweep gas flow rate, 2 (arbitrary units); aux gas heater temperature, 400 °C; capillary temperature, 275 °C. The instrument was calibrated using a positive ESI calibration solution prepared according to the operating manual. All samples were analyzed using two alternating experiment types: full scan mode from *m/z* 60–500 at a resolving power of 70,000 and with a 1 × 10^6^ automatic gain control (AGC) target and a data independent acquisition (DIA) experiment with an inclusion list at a resolving power of 35,000 and with a 5 × 10^5^ AGC target. The collision energy (CE) was set between 10 and 40 V.

### 3.4. Sample Derivatization

Two microliters of a test sample were added to an Eppendorf tube containing 0.984 mL of acetone. Then, 10 µL of an aqueous solution of 100 mg/mL potassium carbonate and 4 µL of IBCF were added to the Eppendorf tube. The reaction mixture was stirred for 5 min at ambient temperature in a thermomixer, and then 2 µL of the reaction mixture were diluted by 1:500 with aqueous 0.1% formic acid prior to LC–MS/MS analysis.

## 4. Conclusions

Herein, we report the first development and validation of a specific, accurate, precise, and robust analytical method to quantitate TRIS by RP-LC-ESIMS/MS after derivatization in a complex, virus-containing, vaccine formulation. This novel approach was used to determine the TRIS concentration in the IIBR COVID-19 BriLife^®^ vaccine formulation. The high performance of the method stems from combining chromatographic separation with MS/MS MRM, resulting in inherent sensitivity and selectivity. Moreover, the derivatization reaction improved the peak shape and retention time of the chromatogram. Elimination of the matrix effect and interfering peaks was achieved by a high sample dilution. Thus, the validated GMP-compliant RP-LC-ESI–MS/MS-based method presented herein can quantify TRIS concentration in a complex cell-derived matrix and can therefore be adapted for the determination of TRIS in other vaccine and pharmaceutical formulations, as well as in biological fluids, such as urine and plasma.

## Figures and Tables

**Table 1:** Accuracy summary.

Added TRIS	Spiking Level	Expected Conc. (mg/mL)	Obtained Conc. (mg/mL)	Recovery *
0%	50%	1.24 ± 0.07	1.30 ± 0.12	105%
50%	75%	1.86 ± 0.05	1.72 ± 0.13	92%
100%	100%	2.35 ± 0.14	2.42 ± 0.18	103%
150%	125%	3.06 ± 0.12	2.92 ± 0.08	95%

Each TRIS concentration result is the mean ± 1 standard deviation (SD) of three independent derivatizations from a vaccine preparation. * Recovery = [Obtained conc.]/[Expected conc.]

**Table 2:** Repeatability summary.

		Method		Instrument	
		Conc. (mg/mL)	Rt (min.)	Conc. (mg/mL)	Rt (min.)
Experiment 1	Mean	2.33	5.98	2.40	5.99
	SD	0.12	0.01	0.03	0.01
	%CV	5.2%	0.09%	1.2%	0.09%
Experiment 2	Mean	2.51	5.98	2.50	5.98
	SD	0.30	0.004	0.03	0.00
	%CV	11.9%	0.07%	1.2%	0.00%
Experiment 3	Mean	2.25	5.99	2.25	5.99
	SD	0.23	0.004	0.04	0.005
	%CV	10.4%	0.07%	1.7%	0.09%

**Table 3:** Intermediate precision of TRIS concentrations for a vaccine preparation and the QC sample.

	Conc. in Vaccine (mg/mL)	Conc. in QC Sample (mg/mL)
Experiment 1	2.33 ± 0.12	2.44 ± 0.26
Experiment 2	2.50 ± 0.30	2.39 ± 0.11
Experiment 3	2.25 ± 0.23	2.27 ± 0.15
Average	2.36 ± 0.13	2.37 ± 0.09
%CV	5.6%	3.8%
%RE ^a^	−1.7%	−1.4%

^a^ Deviation from the expected (weighed) 2.4 mg/mL TRIS in the formulation. Each concentration result is the mean ± 1 SD of at least three independent derivatizations. The intermediate precision, i.e., the %CV of the TRIS concentration, was calculated among the three different work days and two different analysts.

**Table 4:** Range analysis expressed as repeatability at 75%−125% of the TRIS target concentration.

TRIS Conc. (mg/mL)	Distance from Target Conc.	%CV ^a^	%CV ^b^
1.8	75%	8.8%	9.3%
2.4	100%	10.1%	9.5%
3.0	125%	N.A.	6.6%
3.6	150%	6.9%	N.A.

**^a^** Inferred from linearity studies. The %CV was calculated by the root mean square approach using data from five independent experiments. Measurements were performed in triplicate for each experiment. **^b^** Inferred from accuracy studies. The %CV was calculated by the root mean square approach using data from triplicates of three independent spiking procedures.

**Table 5:** Robustness data.

	Normal	Low Column Temp.	High Column Temp.	Low Flow Rate	High Flow Rate	2nd Column
Average peak area	8.1 × 10^5^	8.2 × 10^5^	7.8 × 10^5^	8.0 × 10^5^	7.8 × 10^5^	7.4 × 10^5^
Peak area SD	2.6 × 10^4^	1.0 × 10^4^	2.1 × 10^4^	1.9 × 10^4^	1.4 × 10^4^	3.8 × 10^4^
%CV	3.2%	2.3%	2.7%	2.4%	1.8%	5.2%
%RE ^a^	-	2.0%	−2.8%	−0.4%	−2.8%	−8.1%

^a^ the deviation from the average peak area of the TRIS signal under normal conditions. The TRIS quantitation robustness was evaluated by calculating the %CV and %RE following slight modifications in the column temperature and flow rate and by using a column from a different batch.

**Table 6:** System suitability summary.

Experiment	%CV ^a^	%RE ^b^
1	1.1%	3.1%
2	2.8%	5.9%
3	2.2%	3.4%
4	3.4%	0.5%
5	2.7%	0.6%

^a^ Repeatability of the peak area results for the first six injections. ^b^ Difference between the peak area obtained for the seventh injection at the end of the day and the mean peak area obtained for the first six injections.

## Data Availability

The data presented in this study supporting the results are available in the main text and Supplementary Materials. Additional data are available upon request from the corresponding authors.

## Supplementary Materials

The following supporting information can be downloaded at: https://www.mdpi.com/article/10.3390/molecules28010073/s1, Figure S1. Fragmentation pattern of protonated TRIS-IBCF and plausible ion structures. Table S1. Method repeatability. Table S2. Instrument repeatability. Table S3. Robustness data. Table S4. System suitability.

## References

[1] Yahalom-Ronen Y., Tamir H., Melamed S., Politi B., Shifman O., Achdout H., Vitner E.B., Israeli O., Milrot E., Stein D. (2020). A single dose of recombinant VSV-∆G-spike vaccine provides protection against SARS-CoV-2 challenge. Nat. Commun..

[2] Makovitzki A., Jayson A., Oren Z., Lerer E., Kafri Y., Dor E., Cherry L., Tzadok H., Levin L., Hazan O. (2021). In-line monitoring of downstream purification processes for VSV based SARS-CoV-2 vaccine using a novel technique. BioTech.

[3] Lerer E., Oren Z., Kafri Y., Adar Y., Toister E., Cherry L., Lupu E., Monash A., Levy R., Dor E. (2021). Highly efficient purification of recombinant VSV-∆G-Spike vaccine against SARS-CoV-2 by flow-through chromatography. BioTech.

[4] Gomori G. (1955). Preparation of buffers for use in enzyme studies. Methods Enzymol..

[5] Windholz M. (1983). The Merck Index.

[6] Nahas G.G., Sutin K.M., Fermon C., Streat S., Wiklund L., Wahlander S., Yellin P., Brasch H., Kanchuger M., Capan L. (1998). Guidelines for the treatment of acidaemia with THAM. Drugs.

[7] Advising Individuals with Allergies on Their Suitability for COVID-19 Vaccine Moderna. https://uat.sps.nhs.uk/articles/advising-individuals-with-allergies-on-their-suitability-for-covid-19-vaccine-moderna/.

[8] Vaccine Information Fact Sheet for Recipients and Varegivers, ModernaTX, Inc. https://eua.modernatx.com/covid19vaccine-eua/bivalent-dose-recipient.pdf.

[9] Hatziantoniou S., Maltezou H.C., Tsakris A., Poland G.A., Anastassopoulou C. (2021). Anaphylactic reactions to mRNA COVID-19 vaccines: A call for further study. Vaccine.

[10] European Medicines Agency (2021). CHMP Assessment Report on Group of an Extention of Marketing Authorization and Variations—Comirnaty.

[11] Pfizer-Biontech COVID-19 Vaccine: Fact Sheet for Healthcare Providers Administering Vaccine (Vaccination Providers), Emergency Use Authorization (EUA) of the Pfizer-Biontech COVID-19 Vaccine to Prevent Coronavirus Disease 2019 (COVID-19). https://labeling.pfizer.com/ShowLabeling.aspx?id=14471.

[12] Hall R.E., Havner G.D., Good R., Dunn D.L. (1995). Ion chromatographic method for rapid and quantitative determination of tromethamine. J. Chromatogr. A.

[13] Gumbhir K., Mason W.D. (1992). High-performance liquid chromatographic method for the determination of tris (hydroxymethyl) aminomethane (tromethamine) in human plasma. J. Chromatogr. B Biomed. Sci. Appl..

[14] McArdle F.A., Meehan C.J. (1998). Determination of tromethamine in an eye-care pharmaceutical by capillary electrophoresis. Analyst.

[15] Guo Y., Huang A. (2003). A HILIC method for the analysis of tromethamine as the counter ion in an investigational pharmaceutical salt. J. Pharm. Biomed. Anal..

[16] Rodríguez S.A., Qiu F., Mulcey M., Weigandt K., Tamblyn T. (2015). Monitoring the chemical and physical stability for tromethamine excipient in a lipid based formulation by HPLC coupled with ELSD. J. Pharm. Biomed. Anal..

[17] (2017). European Pharmacopoeia (Ph. Eur.).

[18] US Pharmacopeia (2016). United States Pharmacopeia National Formulary, Official Monographs. Tromethamine.

[19] Morris M.J., Hsieh J.Y.-K. (1993). Determination of tris(hydroxymethyl)aminomethane (tromethamine) in human plasma and urine by high-performance liquid chromatography with fluorescence detection. J. Chromatogr..

[20] Naidong W., Ghodbane S. (1999). Development and validation of an HPLC method for the quantitation of tromethamine in iopamidol injection. J. Liq. Chromatogr. Relat. Technol..

[21] Beck T.I.H., Toussaint B., Surget E., Herrenknecht C., Boudy V., Jaccoulet E. (2022). Investigation of hydrophilic interaction liquid chromatography coupled with charged aerosol detector for the analysis of tromethamine. Talanta.

[22] Shamai Yamin T., Prihed H., Weissberg A. (2019). Challenges in the identification process of phenidate analogues in LC-ESI-MS/MS analysis: Information enhancement by derivatization with isobutyl chloroformate. J. Mass Spectrom.

[23] European Medicines Agency (1995). ICH Topic Q 2 (R1)—Validation of Analytical Procedure: Text and Methodology.

[24] Stolker A.A.M., Stephany R., van Ginkel L. (2000). Identification of residues by LCMS. The application of new EU guidelines. Analusis.

[25] Cino J., Mirro R., Kedzierski S. (2011). An Update on the Advantages of Fibra-Cel® Disks for Cell Culture. https://www.eppendorf.com/uploads/media/Application_bioprocess_shakers_incubators_Application-Note-Boo.pdf.

