# Supplementary material for: Development and Validation of an Innovative Analytical Approach for the Quantitation of Tris(Hydroxymethyl)Aminomethane (TRIS) in Pharmaceutical Formulations by Liquid Chromatography Tandem Mass Spectrometry
Source: Molecules. 2022 Dec 22;28(1):73. doi: 10.3390/molecules28010073 (PMC9822354; doi:10.3390/molecules28010073)
Supplement: Supplementary file 1 [file molecules-28-00073-s001.zip › molecules-2067575-supplementary.pdf]

Manuscript supplementary tables

Table S1. Method repeatability.

	Experiment 1		Experiment 2		Experiment 3	
	Conc. (mg/ml)	Rt (min)	Conc. (mg/ml)	Rt (min)	Conc. (mg/ml)	Rt (min)
**Derivate 1**	2.40	5.99	2.27	5.98	2.49	5.99
**Derivate 2**	2.50	5.99	2.42	5.98	2.28	5.99
**Derivate 3**	2.28	5.98	2.08	5.98	2.28	5.99
**Derivate 4**	2.15	5.98	2.85	5.98	2.07	5.99
**Derivate 5**	2.29	5.98	2.67	5.99	2.47	5.98
**Derivate 6**	2.36	5.98	2.74	5.98	1.89	5.99

Table S2. Instrument repeatability.

	Experiment 1		Experiment 2		Experiment 3	
	Conc. (mg/ml)	Rt (min)	Conc. (mg/ml)	Rt (min)	Conc. (mg/ml)	Rt (min)
**Run 1**	2.38	5.98	2.54	5.98	2.24	5.99
**Run 2**	2.39	5.98	2.49	5.98	2.23	5.98
**Run 3**	2.38	5.99	2.49	5.98	2.25	5.99
**Run 4**	2.45	5.99	2.52	5.98	2.29	5.98
**Run 5**	2.38	5.99	2.45	5.98	2.19	5.99
**Run 6**	2.40	5.99	2.51	5.98	2.29	5.98

Table S3. Robustness data.

	Normal	Low column temp.	high column temp.	Low flow rate	High flow rate	2nd column
**Run1**	8.0E+05	8.4E+05	8.0E+05	7.9E+05	7.8E+05	7.0E+05
**Run2**	8.5E+05	8.1E+05	7.9E+05	8.2E+05	8.0E+05	7.1E+05
**Run3**	7.8E+05	7.9E+05	7.6E+05	7.7E+05	7.8E+05	7.2E+05
**Run4**	8.1E+05	8.2E+05	7.7E+05	8.3E+05	7.7E+05	7.7E+05
**Run5**	8.1E+05	8.3E+05	7.6E+05	8.0E+05	7.7E+05	8.0E+05
**Run6**	7.8E+05	8.4E+05	8.2E+05	8.1E+05	7.9E+05	7.4E+05

Table S4. System suitability. One representative experiment.

Injection	peak area (counts)	Mean (counts)	%CV [a]	%RE [b]
**1**	7.14E+05			
**2**	7.19E+05			
**3**	7.15E+05			
**4**	7.19E+05	7.16E+05	1.13	
**5**	7.01E+05			
**6**	7.25E+05			
**7**	6.94E+05			3.05

[a] repeatability of peak area results of first six injections

[b] peak area difference between a seventh injection at the end of the day and the mean of the first six injections

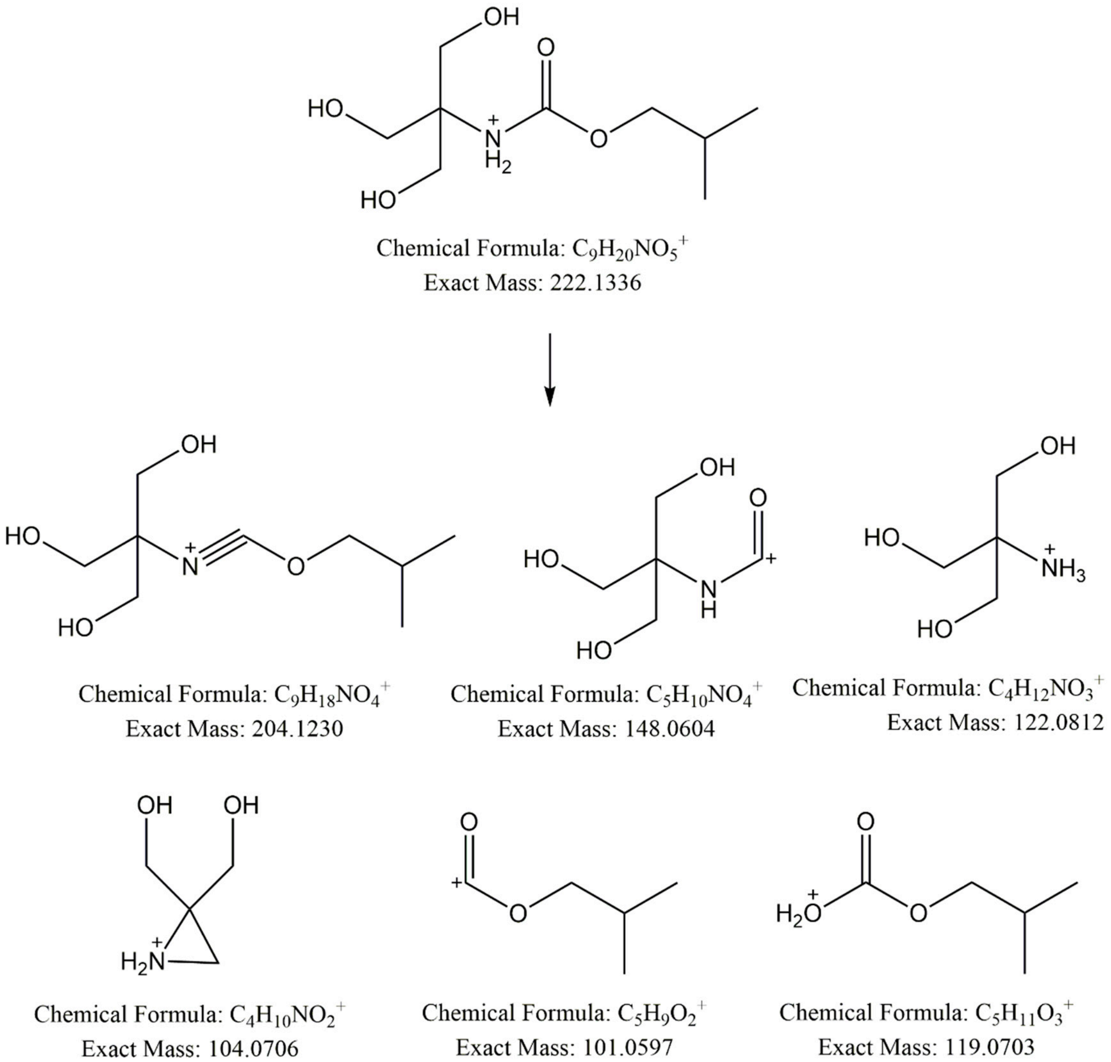

Figure S1. Fragmentation pattern of protonated TRIS-IBCF and plausible ion structures.
